# Neurofibromatosis 2, radiosurgery and malignant nervous system tumours

**DOI:** 10.1054/bjoc.1999.1030

**Published:** 2000-02

**Authors:** M E Baser, D G R Evans, R K Jackler, E Sujansky, A Rubenstein

**Affiliations:** Los Angeles, USA; Department of Medical Genetics, St Mary’s Hospital, Manchester, UK; Departments of Otolaryngology and Neurological Surgery, University of California at San Francisco, San Francisco, CA, USA; Division of Genetic Services, University of Colorado Health Sciences Center/Children’s Hospital, Denver, CO, USA; Department of Neurology, Mt Sinai School of Medicine, New York, NY, USA


*British Journal of Cancer* (2000) **82**(4), 998
© 2000 Cancer Research Campaign
DOI: 10.1054/ bjoc.1999.1030, available online at http://www.idealibrary.com on IDEAL®

Letter to the Editor

### Neurofibromatosis 2, radiosurgery and malignant nervous system tumours

**Sir**

Neurofibromatosis 2 (NF2) is a rare (1:40 000) autosomal dominant disease that is caused by mutations of the *NF2* tumour suppressor gene. NF2 is characterized by benign nervous system tumours such as vestibular schwannomas (VSs), intracranial meningiomas and spinal tumours. Kondziolka et al (1998) reported the outcomes of radiosurgery for unilateral sporadic VSs, but NF2 patients were excluded and follow-up was limited. The consequences of radiosurgery for histologically benign NF2 tumours merit study due to the mutagenic potential of ionizing radiation. Somatic mutation could contribute to the transformation or acceleration of existing tumours, and to the development of secondary tumours, because NF2 patients have an inactivated germ-line *NF2* allele. Ionizing radiation is known to have such effects in hereditary retinoblastoma (Wong et al, 1997). We conducted this study because the prevalence of, and risk factors for, malignant nervous system tumours in NF2 are unknown.

We surveyed genetics, otolaryngology and neurology/neurosurgery centres in North America and Europe with a total of 1348 NF2 patients. There were nine malignant nervous system tumours in the estimated 1242 NF2 patients who did not have previous radiosurgery. This prevalence of 725 per $10^5$ (95% confidence interval (CI) 253–1197 per $10^5$) is significantly greater than the population prevalence of 1.13 per $10^5$ (95% CI 1.09–1.15 per $10^5$) (SEER Program Public-Use CD-ROM, 1998). There were five malignant peripheral nerve sheath tumours (MPNSTs), two malignant meningiomas, one malignant ependymoma and one anaplastic astrocytoma (median age at tumour diagnosis, 14 years; range 7–35 years). The population prevalences of these tumours are MPNST, 0.00 per $10^5$; malignant meningioma, 0.42 per $10^5$; malignant ependymoma, 0.05 per $10^5$; and anaplastic astrocytoma, 0.66 per $10^5$ (SEER Program Public-Use CD-ROM, 1998). Thus, NF2 patients have a significantly increased prevalence of MPNST (402 per $10^5$; 95% CI 50–754 per $10^5$).

After radiosurgery for benign tumours, five NF2 patients developed malignant tumours in high-dose areas (three MPNSTs, one malignant meningioma and one malignant ependymoma; median age at tumour diagnosis, 32 years). In three large NF2 patient series that were included in this study, 47 of 599 patients (7.8%) received radiosurgery (DGR Evans et al, unpublished data). By extrapolation, about 106 of the 1348 patients in this study would have received radiosurgery, suggesting that malignant transformation in five of 106 patients was radiation-associated (4717 per $10^5$; 95% CI 681–8753 per $10^5$).

Radiosurgery is typically used for NF2 patients with aggressive tumours, or who refuse surgery, or who are poor surgical risks or elderly. The results of this study should not be used to interdict radiosurgery for these patients; in addition, the results of this study reflect past exposures and lower doses of ionizing radiation are used in current radiosurgery protocols. However, observation without surgery, decompression without tumour removal, and surgical excision are the preferred therapies for other NF2 patients, particularly those who are young.

### ACKNOWLEDGEMENTS

We thank the NF2 patients for their participation and Dr JM Friedman for many helpful comments.

*ME Baser*[1], *DGR Evans*[2], *RK Jackler*[3], *E Sujansky*[4] *and A Rubenstein*[5]

*[1]Los Angeles, USA, [2]Department of Medical Genetics, St Mary's Hospital, Manchester, UK, [3]Departments of Otolaryngology and Neurological Surgery, University of California at San Francisco, San Francisco, CA, USA, [4]Division of Genetic Services, University of Colorado Health Sciences Center/Children's Hospital, Denver, CO, USA, [5]Department of Neurology, Mt Sinai School of Medicine, New York, NY, USA*

### REFERENCES

Kondziolka D, Lunsford LD, McLaughlin MR and Flickinger JC (1998) Long-term outcomes after radiosurgery for acoustic neuroma. *N Engl J Med* **339**: 1426–1433

Surveillance, Epidemiology and End Results (SEER) Program Public-Use CD-ROM (1973–1995), National Cancer Institute, DCPC, Surveillance Program, Cancer Statistics Branch, released April 1998, based on the August 1997 submission. Rates are for 1986–1995 data age-adjusted to the 1980 US standard

Wong FL, Boice JD Jr, Abramson DH et al (1997) Cancer incidence after retinoblastoma: radiation dose and sarcoma risk. *JAMA* **278**: 1262–1267

